# Tryptophan Metabolism Regulates Proliferative Capacity of Human Pluripotent Stem Cells

**DOI:** 10.1016/j.isci.2021.102090

**Published:** 2021-01-26

**Authors:** Shota Someya, Shugo Tohyama, Kotaro Kameda, Sho Tanosaki, Yuika Morita, Kazunori Sasaki, Moon-Il Kang, Yoshikazu Kishino, Marina Okada, Hidenori Tani, Yusuke Soma, Kazuaki Nakajima, Tomohiko Umei, Otoya Sekine, Taijun Moriwaki, Hideaki Kanazawa, Eiji Kobayashi, Jun Fujita, Keiichi Fukuda

**Affiliations:** 1 Department of Cardiology, Keio University School of Medicine, Shinjuku, Tokyo 160-8582, Japan; 2 Department of Organ Fabrication, Keio University School of Medicine, Shinjuku, Tokyo 160-8582, Japan; 3 Endowed Course for Severe Heart Failure Treatment II, Keio University School of Medicine, Shinjuku, Tokyo 160-8582, Japan; 4 Human Metabolome Technologies, Inc., Tsuruoka, Yamagata 997-0052, Japan

**Keywords:** Cell Biology, Metabolomics, Stem Cell Research

## Abstract

Human pluripotent stem cells (hPSCs) have a unique metabolic signature for maintenance of pluripotency, self-renewal, and survival. Although hPSCs could be potentially used in regenerative medicine, the prohibitive cost associated with large-scale cell culture presents a major barrier to the clinical application of hPSC. Moreover, without a fully characterized metabolic signature, hPSC culture conditions are not optimized. Here, we performed detailed amino acid profiling and found that tryptophan (TRP) plays a key role in the proliferation with maintenance of pluripotency. In addition, metabolome analyses revealed that intra- and extracellular kynurenine (KYN) is decreased under TRP-supplemented conditions, whereas N-formylkynurenine (NFK), the upstream metabolite of KYN, is increased thereby contributing to proliferation promotion. Taken together, we demonstrate that TRP is indispensable for survival and proliferation of hPSCs. A deeper understanding of TRP metabolism will enable cost-effective large-scale production of hPSCs, leading to advances in regenerative medicine.

## Highlights

• TRP is the only AA that enables enhanced hPSC proliferation by supplementation
• hPSCs proliferate with pluripotency after long-term culture in TRP supplementation
• The proliferative properties of hPSCs are independent of AhR signaling
• TRP-derived NFK contributes to enhanced hPSC proliferation

## Graphical Abstract

**Figure:**
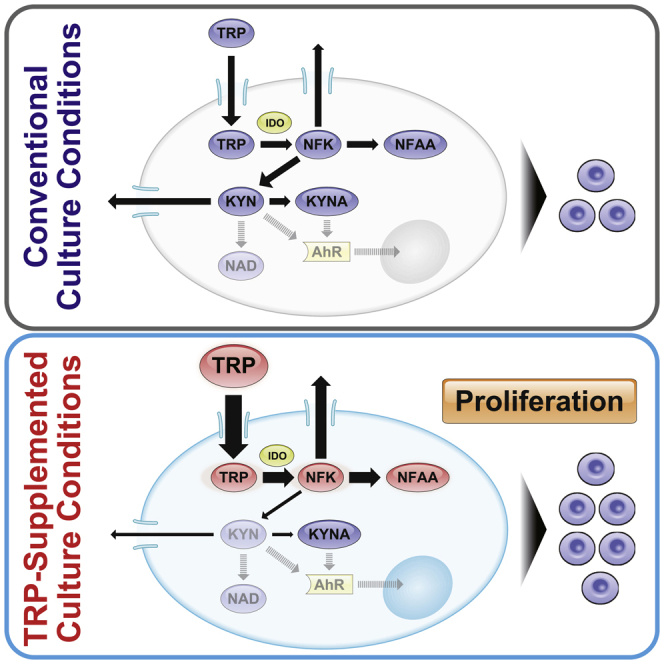

## Introduction

Human pluripotent stem cells (hPSCs), including human embryonic stem cells (hESCs) and human induced pluripotent stem cells (hiPSCs), have the capacity to differentiate into various cell types, making them a promising cell source for regenerative therapy and drug discovery. For clinical applications and industrialization, a large number of cells are needed, which requires large amounts of expensive culture media. To cost-effectively mass-produce cells, hPSCs should be cultured under optimal culture conditions that promote proliferation while maintaining pluripotency.

Metabolism plays a key role in the maintenance of pluripotency and cell survival of hPSCs (Carey et al., 2015; Folmes et al., 2011; Marsboom et al., 2016; Moussaieff et al., 2015; Shiraki et al., 2014; Teslaa et al., 2016; Tohyama et al, 2013, 2016; Vardhana et al., 2019; Wang et al., 2009). hPSCs depend on activated glycolysis and glutamine metabolism for production of ATP, as well as biomass for maintenance of pluripotency and cell survival. Methionine metabolism is also important, because methionine-derived S-adenosylmethionine is a key metabolite for maintaining pluripotency. Understanding the unique metabolism of hPSCs could lead to development of a method for efficient differentiation and elimination of residual undifferentiated stem cells (Marsboom et al., 2016; Shiraki et al., 2014; Tanosaki et al., 2020; Tohyama et al., 2013, 2016; Yanes et al., 2010). However, little is known about optimal concentrations for each amino acid (AA) and the relationship between AA metabolism and proliferation in hPSCs.

We evaluated the consumption profiles and ability of each AA to promote proliferation of hPSCs, focusing on tryptophan (TRP) metabolism. TRP is an essential AA necessary for the integration of protein synthesis. In addition to the production of key metabolites such as serotonin, melatonin, or vitamin B3, it is well known as an important precursor of kynurenine (KYN) and nicotinamide adenine dinucleotide (NAD) stemming from the KYN pathway. The TRP degradation pathway plays an influential role in cancer biology via indoleamine 2,3-dioxygenase (IDO), where KYN acts as a ligand for an aryl hydrocarbon receptor (AhR) to promote tumor progression and metastasis (Opitz et al., 2011). An end product of the KYN pathway, NAD, is known to reduce tumorigenesis by minimizing oncogene-induced DNA damage and subsequent carcinogenesis (Tummala et al., 2014). In hPSCs, although it is known that KYN production is greater for the primed state of hPSCs when compared with the naive state (Sperber et al., 2015), and that *AhR* is minimally expressed in hESCs (Cheng et al., 2015), the functionality of the KYN pathway in hPSCs has yet to be fully elucidated.

Here, we show that TRP metabolism plays a key role in promoting proliferation of hPSCs, without the loss of pluripotency. By utilizing this property, we were able to efficiently obtain a large number of hPSCs under TRP-supplemented culture medium conditions, a technique that could lead to clinical applications and industrialization for hPSCs.

## Results

### TRP supplementation promotes proliferative capacity in hiPSCs

**Figure 1:**
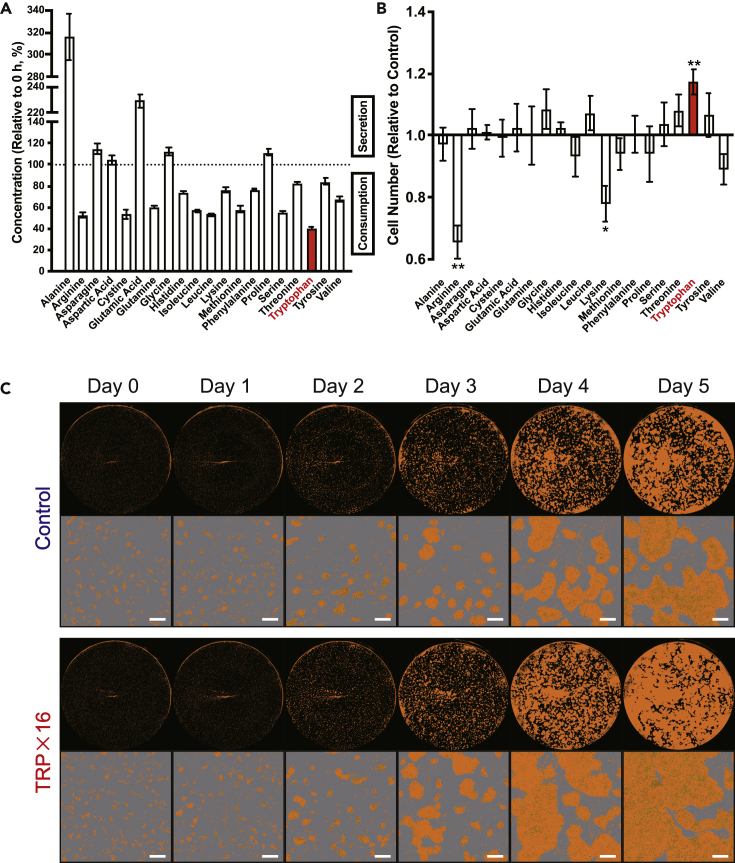
TRP facilitates proliferation of hiPSCs (A) Consumption of all amino acids (AAs) in hiPSCs (201B7) cultured for 72 h, as determined by liquid chromatography-tandem mass spectrometry (LC-MS/MS) (n = 4 independent experiments). (B) Cell counts of hiPSCs (201B7) cultured for 7 days. At day 2, the concentration of AAs was increased to 8-fold of the original concentration of mTeSR1. p values were determined by ratio paired t tests comparing the presence or absence of AA augmentation (n = 6 independent experiments; each control value was derived by the average of quadruplicates within the study). (C) Confluence of hiPSCs (201B7) after 7 days of culture, with 16-fold of the original concentration of tryptophan (TRP) added at 48 h after seeding, versus the control (representative data from n = 6 independent experiments). Scale bar, 500 μm. Data are represented as mean ± SEM; ∗∗p < 0.01.

Consumption of AAs in hiPSCs in mTeSR1 maintenance medium over 3 days was first measured by liquid chromatography-tandem mass spectrometry (LC-MS/MS). Consistent with a previous report where AA consumption profiles were evaluated in hESCs under high-glucose DMEM (Tohyama et al., 2016), arginine, cystine, glutamine, and serine were highly consumed. Although aspartate is known to play a key role in the proliferation of cancer cells (Sullivan et al., 2015), no consumption was observed. A previous study has shown that isoleucine, leucine, and methionine are also highly consumed (Shiraki et al., 2014). Interestingly, among all AAs tested in our study, TRP was the most consumed (Figure 1A). We then tested whether AAs have a proliferative effect on hiPSC growth by adding AAs in increments up to 8-fold of the original concentration. We found that TRP was the only AA that caused significant cell growth 5 days post-exposure, in contrast to other AAs such as arginine and lysine, where cell growth was reduced significantly (Figure 1B). Imaging with IncuCyte (Essen BioScience) showed consistent results, and confluence was robustly increased after exposure to incremented TRP concentrations (Figures 1C and S1A).

### hPSCs continue to proliferate and maintain pluripotency after long-term culture under TRP-supplemented medium

**Figure 2:**
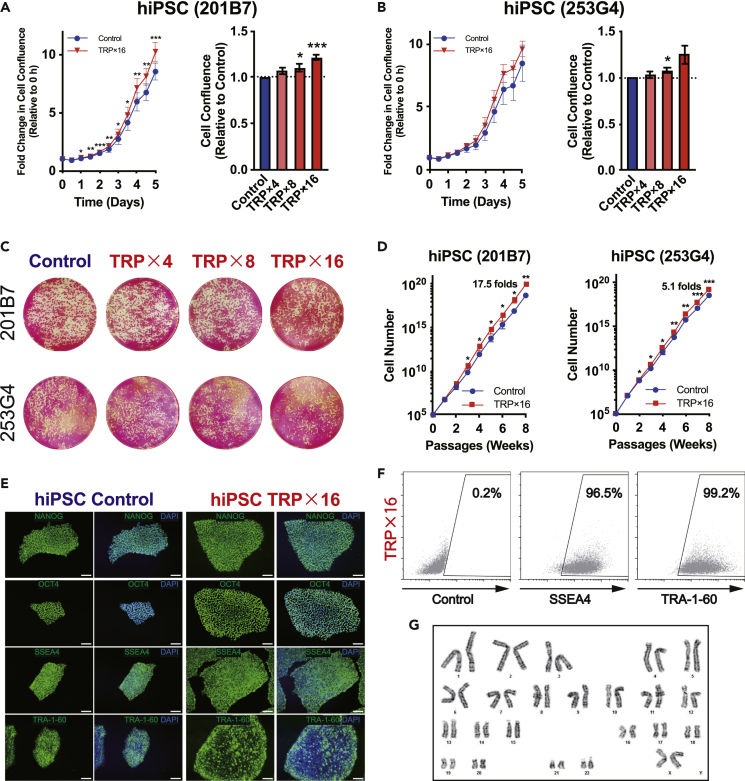
TRP-supplemented medium allows long-term proliferation of hiPSCs with superior efficiency without compromising pluripotency and karyotype (A and B) Cell confluence after 7 days of hiPSC (201B7 and 253G4) culture, with varying folds of TRP concentration added at 48 h after seeding. p values were determined by ratio paired t test (left) and unpaired t test (right) (n = 6 independent experiments for 201B7; n = 3 independent experiments for 253G4 cell lines). (C) Alkaline phosphatase staining of hiPSCs (201B7 and 253G4) cultured for 7 days, with different folds of TRP concentrations added at day 2. (D) Cumulative growth curve of hiPSCs (201B7 and 253G4) cultured in 16-fold TRP-supplemented medium compared with control medium by cell counts. Both cells were adapted to respective medium for at least 1 week before the experiment. p values were determined by ratio paired t test (n = 5 independent experiments). (E) Immunocytochemistry for NANOG, OCT4, SSEA4, and TRA-1-60, performed on hiPSCs (201B7) after 22 passages of culture with TRP-supplemented medium. Scale bar, 100 μm. (F) Flow cytometry data indicating SSEA4 and TRA-1-60 levels of hiPSCs (201B7) after maintenance in TRP-supplemented medium for 15 passages (representative data from three independent experiments). (G) Normal karyotype analysis of hiPSCs (201B7) cultured in 16-fold TRP-supplemented medium for 4 weeks. Data are represented as mean ± SEM; ∗p < 0.05; ∗∗p < 0.01; ∗∗∗p < 0.001.

Next, we determined the optimal cell growth TRP concentration by determining confluence and cell counts. In multiple hPSC lines consisting of both hiPSCs and hESCs, TRP concentration was increased from 4- to 16-fold, and cell confluence showed a corresponding increase, with the most proliferative cells occurring under treatment with a 16-fold TRP concentration (Figures 2A–2C). This enhanced cell growth was preserved through long-term passage up to 10 weeks, and cumulative cell counts of hiPSCs and hESCs were steadily increased across all cell lines, ranging from an approximately 5- to a 17.5-fold increase (Figures 2D and S2A). Interestingly, unlike in hPSCs, TRP supplementation did not significantly promote proliferation in immortalized cell lines, including HeLa and HEK293T cells (Figures S2B and S2C). The ability to retain pluripotency was assessed by various means, including the following: alkaline phosphatase staining, where cells were exposed to TRP-supplemented medium for 5 days; immunocytochemistry staining for NANOG, OCT4, SSEA4, and TRA-1-60 after at least 20 passages; and flow cytometry analysis for SSEA4 and TRA-1-60 after 15 passages. All these staining methods revealed that long-term culture did not deleteriously affect pluripotency markers (Figures 2E and 2F). The karyotype was normal after 4 weeks of culture with treated medium (Figure 2G). These results confirm that hPSCs can be effectively and safely cultured in TRP-supplemented medium without losing key features.

### KYN pathway has a pivotal role in proliferation of hiPSCs independent of AhR signaling

**Figure 3:**
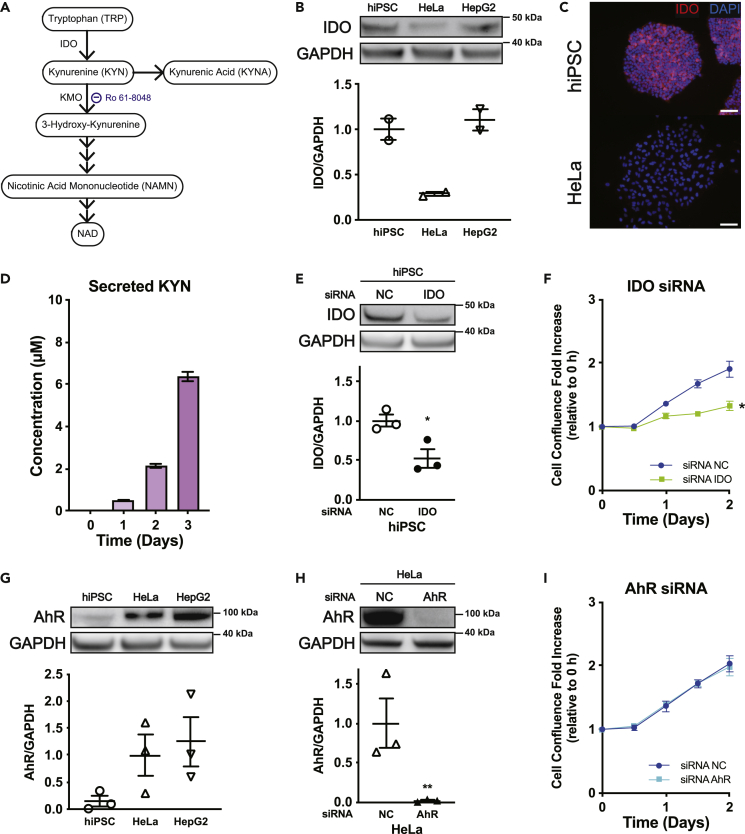
Activation of the KYN pathway via IDO, but not AhR, is crucial for hiPSC survival (A) TRP pathway leading to nicotinamide adenine dinucleotide (NAD) and kynurenic acid (KYNA). IDO, indoleamine 2,3-dioxygenase; KMO, kynurenine 3-monooxygenase. (B) Representative immunoblot protein expressions of IDO in hiPSCs (201B7), HeLa, and HepG2 cells by western blot, and the relative quantified protein expressions of IDO, in which hiPSC levels were set to 1. GAPDH was used as a loading control (n = 2). (C) Immunocytochemistry of IDO in hiPSCs (201B7) and HeLa cells. Scale bar, 100 μm. (D) Serial concentrations of kynurenine (KYN) secreted in medium cultured for 3 days (n = 4 independent experiments). (E) Western blot and the relative protein expression of IDO and GAPDH in hiPSCs (201B7) after *IDO* or negative control (NC) siRNA knockdown. Values were normalized to negative controls. p values were determined by a ratio paired t test (n = 3). (F and I) Proliferation assays of hiPSCs (201B7), performed after seeding 2.5 × 10^5^ cells and 24 h of culture under normal conditions followed by 48-h lipofection with *IDO* or *AhR* (aryl hydrocarbon receptor) siRNA knockdown, comparing against NC siRNA. p values were determined with an unpaired t test (n = 3 independent experiments). (G) AhR levels by western blot and their relative protein expressions were normalized to those of HeLa cells (n = 3). (H) Western blot analysis and the relative protein expressions of AhR and GAPDH in HeLa cells after NC or *AhR* siRNA knockdown. Values were normalized to negative controls. p values were determined with a ratio paired t test (n = 3). Data are represented as mean ± SEM; ∗p < 0.05; ∗∗p < 0.01.

Consistent with previous studies (Shiraki et al., 2014), we found that TRP deprivation halted cell growth and led to cell death, and most hiPSCs were incapable of survival after 2 and 4 days of culture in TRP-depleted custom DMEM and TRP-depleted StemFit maintenance medium, respectively (Figure S3A), confirming that TRP is essential in cell growth of hiPSCs and hESCs. To elucidate the mechanism for enhanced proliferation of hPSCs, we first attempted to determine if the KYN pathway has a pivotal role in hPSC proliferation, which is known to lead to the production of KYN, a major ligand for AhR (Opitz et al., 2011), as well as NAD, an important final metabolite of TRP involved in various biochemical reactions, such as glycolysis and oxidative phosphorylation (Purushotham et al., 2009; Rodgers et al., 2005) (Figure 3A). Western blot and immunocytochemistry analysis revealed that hiPSCs have higher IDO expression compared with human cervical cancer (HeLa) cells (Figures 3B and 3C), or differentiated cells, including hiPSC-derived cardiomyocytes and human dermal fibroblasts (Figure S3B). These results were verified by an LC-MS/MS analysis, which demonstrated that KYN was secreted after 3 days of normal maintenance culture (Figure 3D). Knockdown of *IDO* by small interfering RNA (siRNA) resulted in a reduction of hiPSC cell growth (Figures 3E and 3F), demonstrating that activated IDO is crucial for cell growth of hiPSCs. Next, we tested if abundant expression of AhR plays a role in cell proliferation. In contrast to cancer cell lines, including HeLa, HepG2, and HEK293T cells, AhR expression was scarce in hiPSCs (Figure 3G) and neither its inhibition by siRNA or potent inhibitor StemRegenin-1 (SR-1) influence cell growth capability (Figures 3H, 3I, and S3C) nor did an increase in growth occur with the addition of a potent AhR ligand, 2,3,7,8-tetrachlorodibenzo-p-dioxin (TCDD) (Figure S3D). To analyze the pathway leading to NAD *de novo* synthesis, we first blocked KYN 3-monooxygenase (KMO), a KYN-catalyzing enzyme and important determinant of NAD production, with its potent inhibitor Ro 61-8048, which did not affect cell survival or proliferation (Figure S3E). Addition of a nicotinic acid mononucleotide (a substrate preceding NAD synthesis) did not amplify cell growth (Figure S3F). Furthermore, an NAD/NADH assay using TRP-depleted or TRP-supplemented custom DMEM demonstrated that 24 h of TRP depletion or supplementation neither significantly altered the total NAD and NADH nor the NAD/NADH ratio (Figure S3G). Finally, to test the role of aspartate, which is crucial for cancer cell proliferation and for the maintenance of an optimal NAD/NADH ratio (Gui et al., 2016), we supplemented hiPSCs with up to a 10 mM concentration of aspartate, which is above physiological concentrations, and observed no proliferative benefit (Figure S3H).

### KYN is decreased and NFK is increased under TRP-supplemented conditions

**Figure 4:**
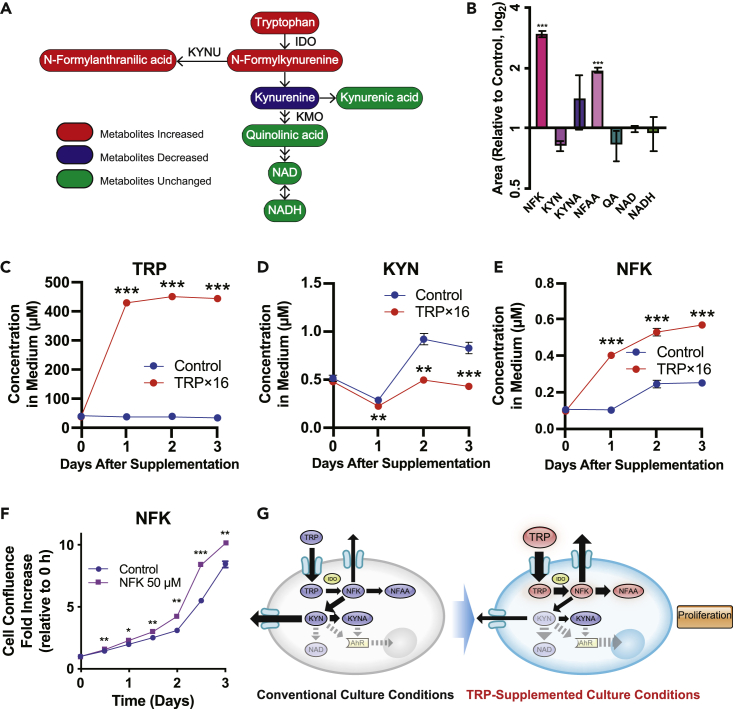
Metabolomic analysis of the TRP pathway in hiPSCs (A) TRP pathway comparing hiPSCs (201B7) incubated with or without TRP-supplemented medium for 24 h, as analyzed by CE-FTMS, where the red or blue coloring indicates increased or decreased metabolites, respectively, and green denotes metabolites that are unchanged (n = 5 replicates). (B) Change in the normalized relative intracellular concentrations of KYN pathway metabolites after TRP exposure for 24 h as determined by CE-FTMS. p values were determined with an unpaired t test, comparing the raw results of each metabolite (n = 5 replicates). NFK, N-formylkynurenine; NFAA, N-formylanthranilic acid; QA, quinolinic acid; others are as mentioned previously. (C–E) Serial extracellular concentrations of TRP, KYN, and NFK, respectively, with or without TRP-supplemented medium for 3 days, analyzed by LC-MS/MS. p values were determined by an unpaired t test (n = 4 replicates). (F) Cell confluence after 5 days of hiPSC (201B7) culture in StemFit maintenance medium, with NFK added at 48 h after seeding. p values were determined by unpaired t test (n = 3). (G) Synoptic diagram indicating the influx of TRP and catabolism of KYN pathway metabolites in hiPSCs, with or without supplementation of TRP-supplemented medium, highlighting diminished change in flux of metabolites distal to NFK. Data are represented as mean ± SEM; p < 0.05; p < 0.01; p < 0.001.

We initially attempted to quantify metabolites of the KYN pathway by utilizing conventional metabolome analysis, by employing capillary electrophoresis time-of-flight mass spectrometry (CE-TOFMS). One drawback of this method is that we could not detect key intermediate metabolites apart from KYN, NAD, and NADH (**data not shown**). In comparison, by applying our newly developed capillary electrophoresis coupled with a Fourier transform mass spectrometry (CE-FTMS) platform to evaluate metabolomics data (Sasaki et al., 2019), we successfully detected a wider range of metabolites, including those of the KYN pathway. We performed a metabolome analysis using CE-FTMS to determine how TRP-supplemented medium influences metabolomics profiles of hiPSCs and compared the metabolites after 24- and 48-h exposure to treated medium. With the exception of isocitrate, metabolites relevant to glycolysis and the tricarboxylic acid cycle did not differ significantly between the conventional and TRP-supplemented media groups at 24 h (Figure S4A). To further determine if glycolysis or oxidative phosphorylation is affected upon TRP metabolism, the flux analyzer was used, which demonstrated no differences in extracellular acidification rate and oxidative consumption rate between the two groups (Figures S4B and S4C). Moreover, hiPSCs contained large amounts of intracellular TRP, following exposure to treated medium (Figures S4D and S4E), with the first-step metabolite, N-formylkynurenine (NFK), increased by up to 3-fold after 24- and 48-h exposure (Figures 4A, 4B, and S4F). Meanwhile, although NFK-derived N-formylanthranilic acid (NFAA) was transiently increased at 24 h, no significant difference was observed at 48 h. Furthermore, KYN demonstrated a general decreased trend (P = 0.0672 at 24 h), whereas the concentration of metabolite quinolinic acid, as well as those of both NAD and NADH, were unchanged up to 24 h. These results agree with those observed in the NAD/NADH assay, despite an unexpected decrease in NAD observed at 48 h (Figures S3G and S4F). The change in flux of TRP metabolites observed by CE-FTMS was consistent with the results obtained from a conventional LC-MS/MS analysis, which demonstrated that whereas secretions of TRP and NFK were consistently elevated, KYN was significantly decreased following TRP supplementation (Figures 4C–4E). An additional metabolome analysis was performed to describe the flux in TRP metabolism by comparing TRP-depleted and TRP-replenished media. Results show that the 6-h depletion leads to significant decreases in TRP, NFK, and KYN, demonstrating that TRP is indispensable in maintaining downstream metabolites of the KYN pathway (Figure S4G). Next, to identify the TRP metabolite responsible for promoting hiPSC proliferation, we assessed the cell proliferation capacity following addition of NFK, which manifested a significant increase in cell confluence (Figure 4F). Taken together, these results imply that TRP, the most consumed AA in hiPSCs, contributes to proliferation when supplemented medium is used, yet this contribution is independent of the catabolism of TRP into KYN and NAD, but rather by increasing the concentration of NFK, thereby bypassing the stimulation of minimally expressed AhR, or upregulation of glycolysis and oxidative phosphorylation by NAD (Figure 4G).

## Discussion

In this study, we evaluated detailed AA profiling in hPSCs and found that TRP was indispensable for survival and proliferation of hPSCs. Consistent with previous studies, arginine, cystine, glutamine, methionine, serine, and TRP were highly consumed AAs, regardless of cell line or culture medium (Tohyama et al., 2016). Recently, some reports have shown that most of these AAs play key roles in the maintenance of pluripotency and survival (Marsboom et al., 2016; Shiraki et al., 2014). However, few studies have examined the role of TRP in hPSC culture maintenance. We demonstrated that TRP supplementation significantly increased proliferative capacity of hPSCs, suggesting that the concentration of TRP in conventional hPSC maintenance media is suboptimal for inducing efficient proliferation. In contrast, supplementation with the other AAs did not promote proliferation, suggesting that concentrations of these AAs are already optimal.

The metabolic signature of hPSCs resembles that of cancer cells in some aspects, as both cells strongly rely on aerobic glycolysis and glutamine oxidation as sources of energy expenditure and biomass production. TRP was shown to be highly consumed in hiPSCs, which was similar to previous findings in cancer cells (Opitz et al., 2011; Snodgrass and Iversen, 1974). Interestingly, TRP supplementation did not directly promote proliferation in cancer cells, whereas it significantly promoted proliferation in hPSCs. In cancer cells, a main purpose of TRP metabolism through IDO is to produce KYN for suppression of antitumor immune T cells via AhR signaling, thus creating a suitable tumor microenvironment that indirectly leads to increased growth (Opitz et al., 2011). Clinically, the level of IDO expressions is known to positively correlate with poor prognosis in some cancers, thus prompting the use of IDO inhibitors, some of which are undergoing clinical trials for cancer chemotherapy (Prendergast et al., 2017). In hiPSCs, IDO was richly expressed, and its inhibition by siRNA knockdown resulted in termination of cell growth, confirming that TRP catabolism is vital for survival of hiPSCs. In contrast, whether AhR signaling is directly associated with cancer cell proliferation remains unclear; nevertheless, it seems to affect proliferative capacity in some cancer cells. For instance, *AhR* knockdown in HepG2 cells was shown to decrease cell proliferation due to downregulation of cell-cycle-related genes (Abdelrahim et al., 2003), whereas agonism of AhR by TCDD was shown to suppress cell proliferation of OVCAR-3, a human ovarian cancer cell line (Li et al., 2014). It was also reported that TRP derivatives negatively regulate cancer cell stemness via AhR signaling, reducing tumorigenicity (Cheng et al., 2015). Aside from cancer cells, immune B cells require functional AhR to optimally proliferate, through activation of cyclin O (Villa et al., 2017). In contrast to cancer cells, we demonstrated that AhR was negligibly expressed in hiPSCs, and its inhibition did not affect cell survival and proliferation, highlighting a distinct signaling property of hPSCs.

To quantify intracellular metabolites under TRP-supplemented conditions or TRP-depleted conditions, we performed CE-FTMS-based metabolome analysis. Conventional CE-TOFMS is a powerful tool to detect both intracellular and extracellular metabolites. However, detection of small amounts of metabolites in the KYN pathway was challenging. To solve this problem, we applied CE-FTMS (Sasaki et al., 2019; Soga and Heiger, 2000; Soga et al, 2002, 2003). Our CE-FTMS-based metabolome analysis showed that supplementation with TRP did not significantly increase the concentration of KYN and kynurenic acid (KYNA), which may also act as an AhR agonist, but significantly increased NFK and NFAA, suggesting that upstream metabolites of KYN contribute to promoting proliferation in hPSCs. These findings support those demonstrating that NFK, rather than its downstream metabolites, are involved in promoting proliferation of hiPSCs.

TRP metabolism also contributes to NAD *de novo* synthesis, which regulates proliferation and metabolism in cancer cells (Tummala et al., 2014). In addition, the presence of NAD is known to be crucial for survival of hiPSCs (Son et al., 2013). However, our study revealed that TRP supplementation did not affect NAD production via the KYN pathway, and TRP deprivation alone did not affect either NAD/NADH ratio or NAD concentration, possibly indicating that NAD production from the salvage pathway or elsewhere is more important than the *de novo* pathway. Thus, TRP-derived NAD does not contribute to promoting survival and proliferation in hPSCs.

Multiple clinical trials in regenerative medicine with hPSCs are currently ongoing. However, the risk of tumorigenicity, as well as prohibitive costs associated with large-scale cell culture, are major barriers to hPSC use in the clinic and industrialization. To address risk of tumorigenicity, we previously developed an innovative method to eliminate undifferentiated stem cells and achieve purification of differentiated cells by altering culture conditions, based on an understanding of metabolic signature in hPSCs (Shiraki et al., 2014; Tanosaki et al., 2020; Tohyama et al., 2013, 2016; Wang et al., 2009). The results of this study suggest that regulation of TRP metabolism promotes proliferative capacity in hPSCs, indicating that large numbers of hPSCs can efficiently be produced at a low cost by improving culture conditions. Understanding unique metabolic signatures of hPSCs will enable advances in regenerative medicine and drug discovery.

### Limitations of the study

Despite the observation that TRP and its downstream metabolite NFK cause increased cell proliferation, the underlying molecular and signaling events regulating cell growth remain to be elucidated, and further study is warranted to strengthen our understanding of a metabolic signature of hPSCs.

### Resources availability

#### Lead contact

Further information and requests for resources and reagents should be directed to and will be fulfilled by the Lead Contact, Shugo Tohyama, Keio University (shugotohyama@keio.jp).

#### Materials availability

This study did not generate new unique reagents.

#### Data and code availability

There is no dataset and/or code associated with the article.

## Methods

All methods can be found in the accompanying Transparent Methods supplemental file.

## Supplemental information

Document S1. Transparent methods and figures S1–S4
