# Supplementary material for: Tryptophan Metabolism Regulates Proliferative Capacity of Human Pluripotent Stem Cells
Source: iScience. 2021 Jan 26;24(2):102090. doi: 10.1016/j.isci.2021.102090 (PMC7878994; doi:10.1016/j.isci.2021.102090)
Supplement: Document S1. Transparent methods and figures S1–S4 [file mmc1.pdf]

**iScience, Volume *24***

## Supplemental Information

# Tryptophan Metabolism Regulates Proliferative Capacity of Human Pluripotent Stem Cells

**Shota Someya, Shugo Tohyama, Kotaro Kameda, Sho Tanosaki, Yuika Morita, Kazunori Sasaki, Moon-Il Kang, Yoshikazu Kishino, Marina Okada, Hidenori Tani, Yusuke Soma, Kazuaki Nakajima, Tomohiko Umei, Otoya Sekine, Taijun Moriwaki, Hideaki Kanazawa, Eiji Kobayashi, Jun Fujita, and Keiichi Fukuda**

# Figure S1

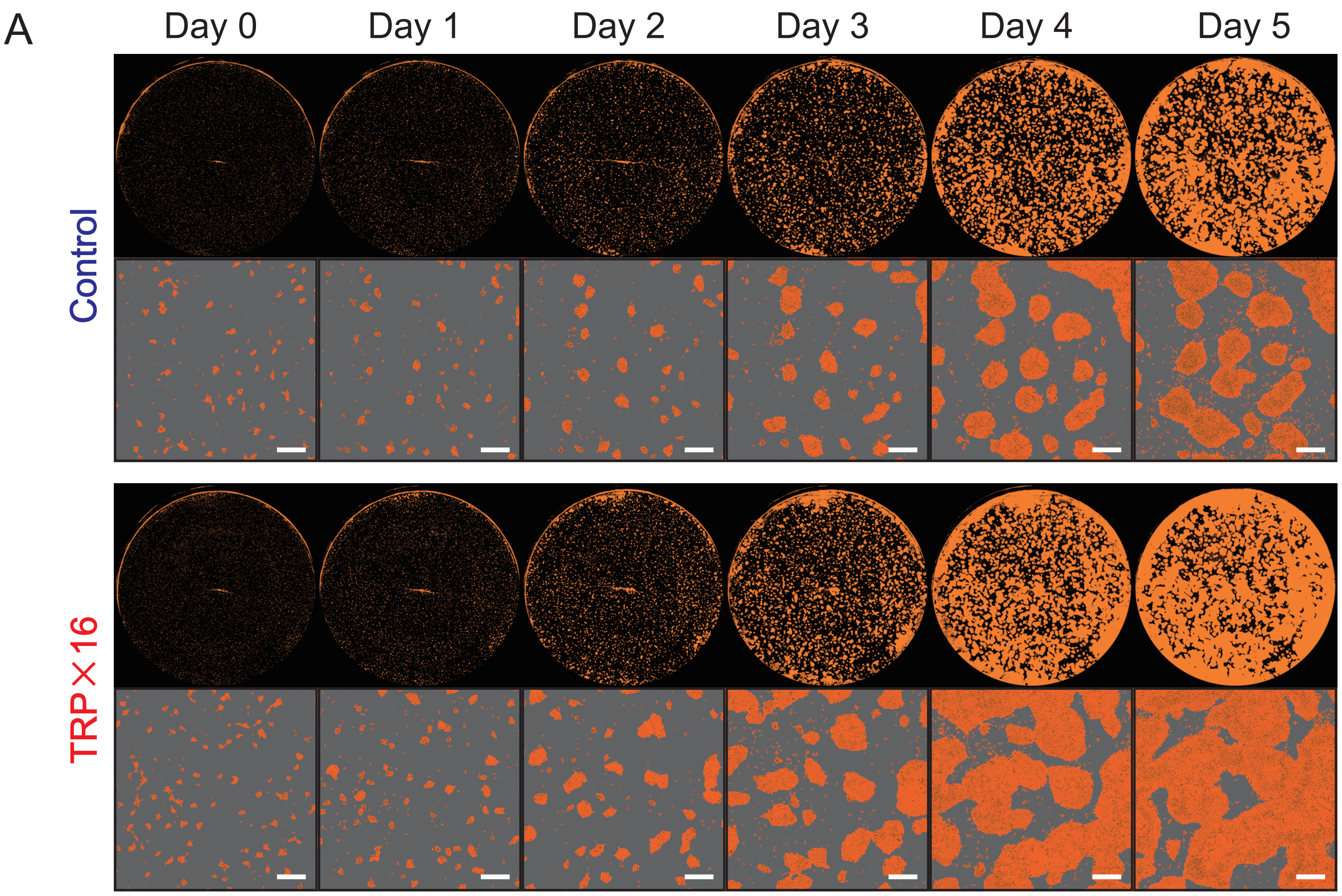

# Figure S2

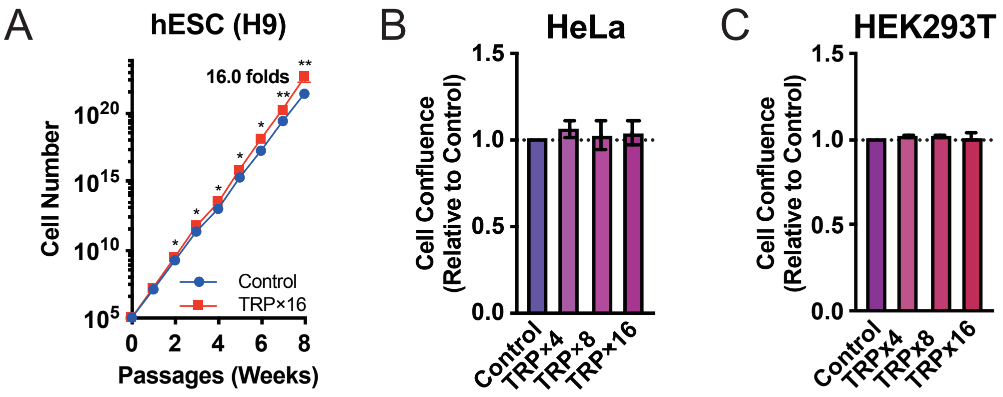
A
hESC (H9)
Cell Number
$10^{20}$
$10^{15}$
$10^{10}$
$10^{5}$
16.0 folds
Control
TRP×16
0 2 4 6 8
Passages (Weeks)
B
HeLa
Cell Confluence (Relative to Control)
1.5
1.0
0.5
0.0
Control
TRP×4
TRP×8
TRP×16
C
HEK293T
Cell Confluence (Relative to Control)
1.5
1.0
0.5
0.0
Control
TRP×4
TRP×8
TRP×16

# Figure S3

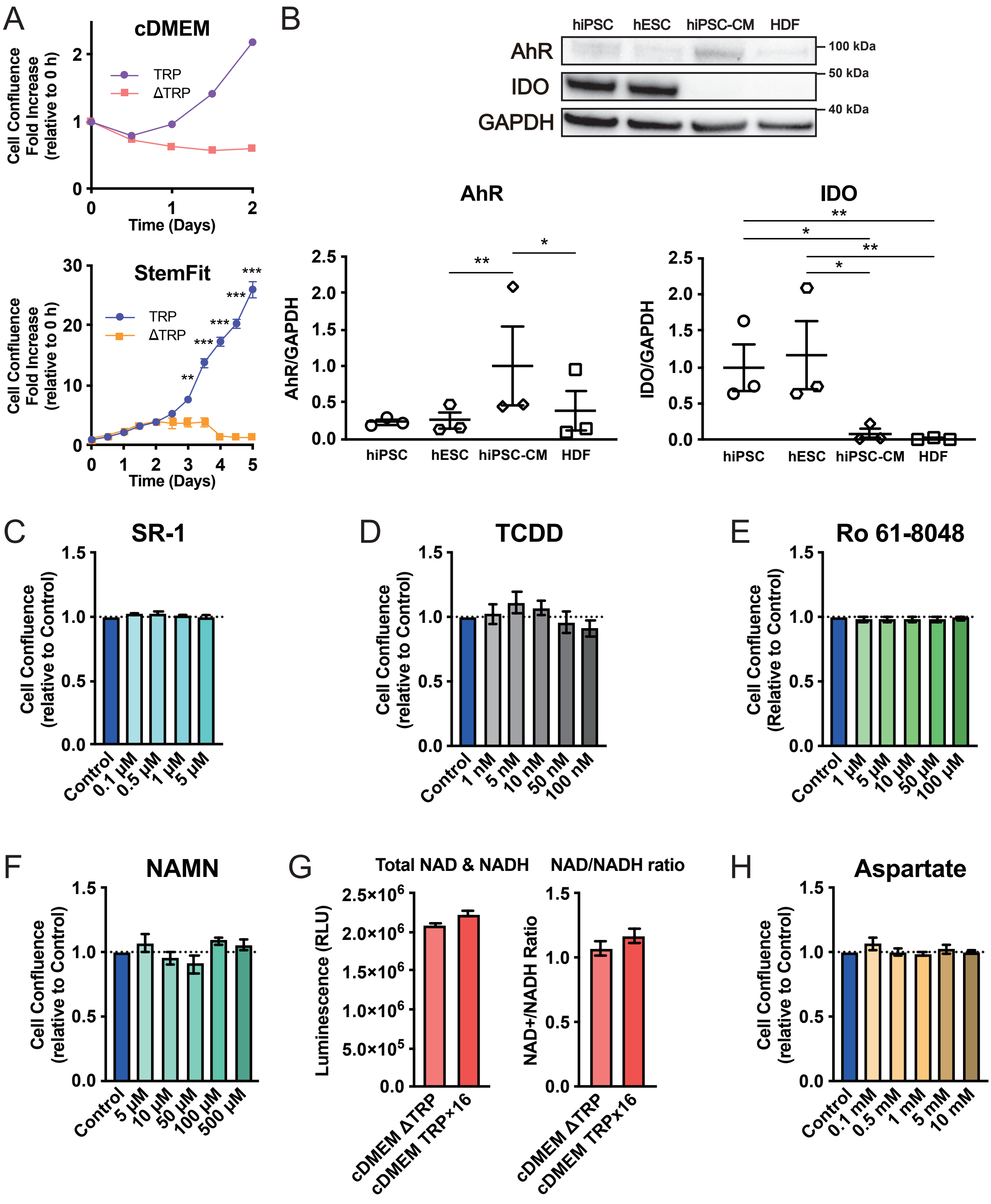
A
cDMEM
Cell Confluence Fold Increase (relative to 0 h)
TRP
ΔTRP
Time (Days)
StemFit
B
hiPSC hESC hiPSC-CM HDF
AhR
IDO
GAPDH
100 kDa
50 kDa
40 kDa
AhR/GAPDH
IDO/GAPDH
C
SR-1
Cell Confluence (relative to Control)
Control 0.1 μM 0.5 μM 1 μM 5 μM
D
TCDD
Control 1 nM 5 nM 10 nM 50 nM 100 nM
E
Ro 61-8048
Cell Confluence (Relative to Control)
Control 1 μM 5 μM 10 μM 50 μM 100 μM
F
NAMN
Control 5 μM 10 μM 50 μM 100 μM 500 μM
G
Total NAD & NADH
Luminescence (RLU)
NAD/NADH ratio
NAD+/NADH Ratio
cDMEM ΔTRP
cDMEM TRP×16
H
Aspartate
Control 0.1 mM 0.5 mM 1 mM 5 mM 10 mM

# Figure S4

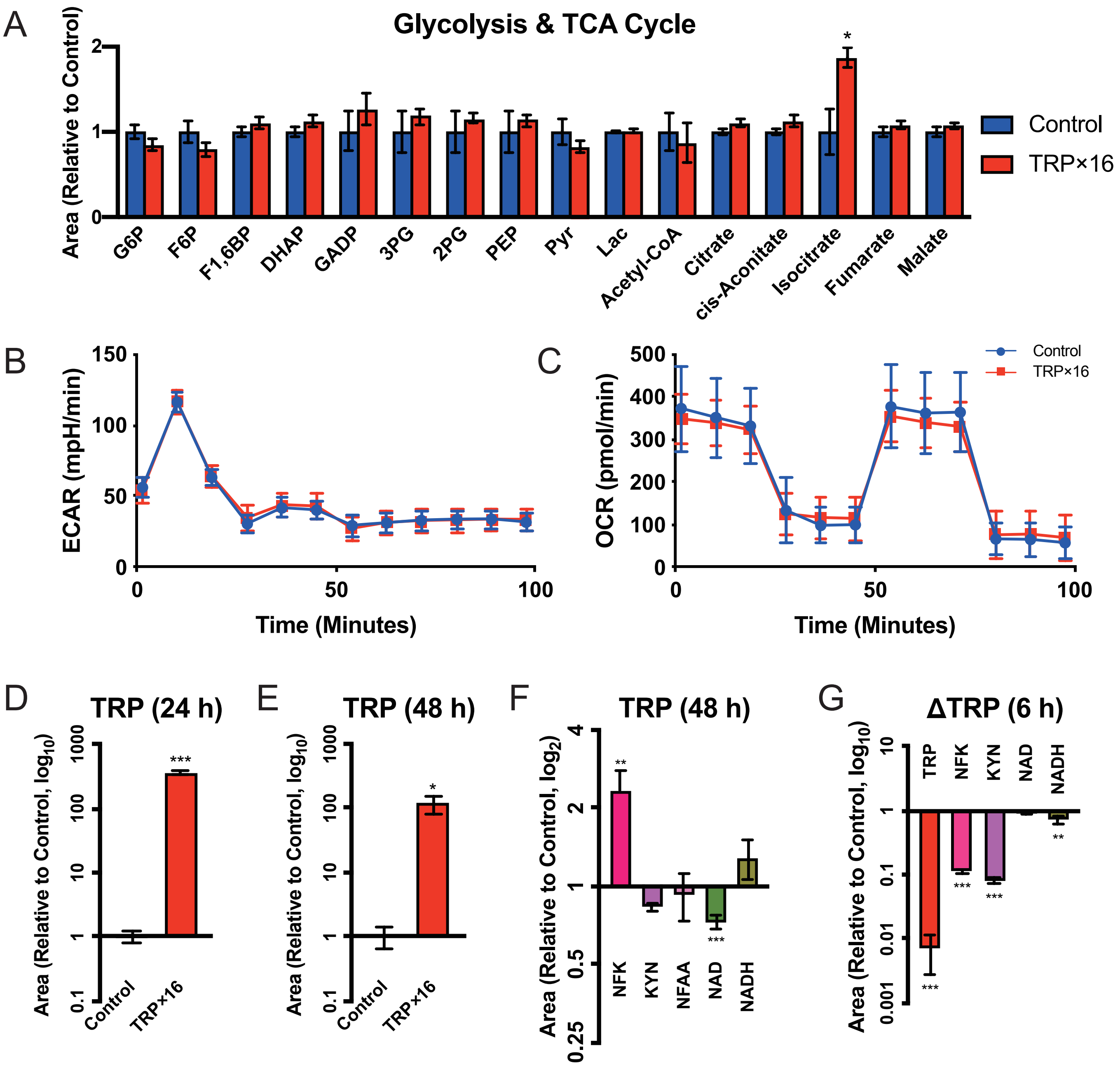
A
Glycolysis & TCA Cycle
Area (Relative to Control)
2
1
0
*
G6P
F6P
F1,6BP
DHAP
GADP
3PG
2PG
PEP
Pyr
Lac
Acetyl-CoA
Citrate
cis-Aconitate
Isocitrate
Fumarate
Malate
Control
TRP×16
B
ECAR (mpH/min)
150
100
50
0
0
50
100
Time (Minutes)
C
OCR (pmol/min)
500
400
300
200
100
0
0
50
100
Time (Minutes)
Control
TRP×16
D
TRP (24 h)
Area (Relative to Control, log10)
1000
100
10
1
0.1
***
Control
TRP×16
E
TRP (48 h)
Area (Relative to Control, log10)
1000
100
10
1
0.1
*
Control
TRP×16
F
TRP (48 h)
Area (Relative to Control, log2)
4
2
1
0.5
0.25
**
***
NFK
KYN
NFAA
NAD
NADH
G
ΔTRP (6 h)
Area (Relative to Control, log10)
10
1
0.1
0.01
0.001
TRP
NFK
KYN
NAD
NADH
***
***
***
**

## SUPPLEMENTARY FIGURE LEGENDS

**Figure S1**. TRP supplementation facilitates cell proliferation in another hiPSC line (Related to Figure 1).

(A) Confluence of hiPSCs (253G4) after 7 days of culture, where 16-fold of the original TRP concentration was added at 48 h after seeding, versus the control (Representative data from n = 3 independent experiments). Scale bar: 500 μm.

**Figure S2**. Influence of TRP supplementation on hESCs and immortalized cell lines, HeLa and HEK293T (Related to Figure 2).

(A) Cumulative growth curve of hESCs (H9) cultured in 16-fold TRP-supplemented medium compared to control medium by cell counts. Cells were adapted to respective medium for at least 1 week prior to the experiment. *P*-values were determined with a ratio paired t-test (n = 5 independent experiments).

(B) Cell confluence after 7 days of HeLa cell culture, with different folds of TRP concentration added at day 2 (n = 3 independent experiments).

(C) Cell confluence after 7 days of HEK293T cell culture, with different folds of TRP concentration added at day 2 (n = 6 replicates).

Data are represented as mean ± S.E.M; *$p < 0.05$; **$p < 0.01$.

**Figure S3**. TRP metabolism regulates proliferative capacity of hPSCs independent of AhR signaling or NAD *de novo* synthesis (Related to Figure 3).

(A) Cell confluence after 4 days of hiPSC (201B7) culture, with custom DMEM (cDMEM) medium, substituted at 48 h after seeding, and cell confluence after 7 days of hiPSC (201B7) culture, with StemFit maintenance medium, substituted at 24 h after seeding. TRP-replenished and TRP-depleted media were denoted as TRP and ΔTRP, respectively. *P*-values were determined by unpaired t-tests (n = 4).

(B) Representative immunoblot protein expressions of aryl hydrocarbon receptor (AhR) and indoleamine 2,3-dioxygenase (IDO) in hiPSCs (253G4), hESCs (H9), hiPSC (253G4)-cardiomyocytes (hiPSC-CMs) and human dermal fibroblasts (HDF) by western blot analysis, and the relative quantified protein expressions of AhR and IDO, in which hiPSC expression levels were normalized to 1. GAPDH was used as a loading control. *P*-values were determined with a ratio paired t-test (n = 3).

(C) Cell confluence after 5 days of hiPSC (201B7) culture, with different concentrations of StemRegenin-1 (SR-1) added at day 1 (n = 2 independent experiments).

(D) Cell confluence after 7 days of hiPSC (201B7) culture, with different concentrations

of 2,3,7,8-Tetrachlorodibenzo-p-dioxin (TCDD) added at day 2 (n = 3 independent experiments).

(E) Cell confluence after 5 days of hiPSC (201B7) culture, with different concentrations of Ro 61-8048 added at day 1 (n = 3 independent experiments).

(F) and (H) Cell confluence after 7 days of hiPSC (201B7) culture, with different concentrations of nicotinic acid mononucleotide (NAMN), or aspartate added at day 2 (n = 3 independent experiments).

(G) Luminescence quantification, indicating total amount of nicotinamide adenine dinucleotide (NAD) and NADH (left) (n = 24 replicates), and the ratio of NAD/NADH (right) (n = 12 replicates), comparing TRP-depleted and TRP-supplemented custom DMEM medium, denoted as cDMEM ΔTRP and cDMEM TRP×16, respectively.

Data are represented as mean ± S.E.M; *$p < 0.05$; **$p < 0.01$; ***$p < 0.001$.

**Figure S4**. Metabolic changes in hiPSCs under TRP-supplemented or depleted conditions (Related to Figure 4).

(A) Metabolome analysis of glycolysis and TCA cycle-associated metabolites comparing hiPSCs (201B7) incubated with or without TRP-supplemented medium for 24 h, as analyzed by CE-FTMS. *P*-values were determined with an unpaired t-test (n = 5 replicates).

(B) and (C) Extracellular acidification rate (ECAR), and oxygen consumption rate (OCR) of hiPSCs (201B7) with or without TRP supplementation (n = 10 replicates).

(D) and (E) Analysis of CE- FTMS showing relative concentration of TRP after exposure to TRP-supplemented medium for 24 and 48 h, respectively, where *P*-values were determined by unpaired t-tests (n = 5 replicates).

(F) Change in the normalized relative intracellular concentrations of KYN pathway metabolites after TRP exposure for 48 h as determined by CE-FTMS. *P*-values were determined with an unpaired t-test, comparing the raw results of each metabolite (n = 5 replicates).

(G) Changes in the normalized relative intracellular concentrations of KYN pathway metabolites after TRP depletion for 6 h as determined by CE-FTMS. *P*-values were determined with an unpaired t-test, comparing the raw results of each metabolite (n = 5 replicates).

Data are represented as mean ± S.E.M; *$p < 0.05$; **$p < 0.01$; ***$p < 0.001$.

## TRANSPARENT METHODS

### Cell Lines

The hiPSC lines (201B7 and 253G4) were provided by the Center for iPS Cell Research and Application, Kyoto University. The ESC line (H9) was provided by WiCELL, and our use complied with the Guidelines on the Distribution and Utilization of Human Embryonic Stem cells, Ministry of Education, Culture, Sports, Science and Technology, Japan. The HEK293T line, HeLa line, and HepG2 line were provided by RIKEN BioResource Research Center, the human dermal fibroblasts line was purchased from Thermo Fisher Scientific, and were maintained in DMEM (Gibco, 11885) supplemented with 10% FBS (Biowest, S1560-500) on 0.1% gelatin scaffolds. Cells were dissociated using 0.25% Trypsin/1 mM EDTA (Nacalai tesque, 35554-64) for passaging. hiPSCs (253G4)-derived cardiomyocytes were obtained and purified using previously described methods (Tohyama et al., 2017; 2016). All cell lines were grown within a humidified 5% $CO_2$ incubator at 37°C, and were regularly tested for Mycoplasma infection.

### Maintenance of hiPSCs or hESCs

hiPSCs were routinely passaged every 7 days. After cells were washed with D-PBS (FUJIFILM Wako Pure Chemical, 045-29795), TrypLE Select (Gibco, 12563011) was applied and cells were incubated at 37°C under 5% $CO_2$ for 3–5 min. Dissociated single cells were collected in growth medium containing mTeSR1 (STEMCELL Technologies, 85850) with 10 μM of CultureSure Y-27632 (FUJIFILM Wako Pure Chemical, 034-24024). Following centrifugation (300 x g for 4 min), supernatant aspiration and addition of growth medium mixture, a cell count was performed with a Vi-CELL XR (Beckman Coulter), and cells were then seeded onto growth factor-reduced Matrigel (Corning, 354230) coated plates. For cumulative cell counts, $1 \times 10^5$ cells were seeded onto a 10 cm dish. Medium was changed every other day using mTeSR1 without Y-27632 unless otherwise stated (Nakagawa et al., 2014). In specified experiments, hiPSCs were maintained with modified StemFit medium, AS103C (Ajinomoto). Karyotypes of hiPSCs were analyzed by Nihon Gene Research Laboratories Inc., Sendai, Japan.

### Preparation of Special Growth Media

Key reagents used were: L-alanine (Sigma, A7469), L-arginine monohydrochloride (Sigma, A6969), L-asparagine monohydrate (Sigma, A7094), L-aspartic acid (Sigma, A7219), L-cysteine hydrochloride monohydrate (Sigma-Aldrich, C6852), L-glutamic acid (Sigma, G8415), L-glutamine (Sigma, G8540), glycine (Sigma, G8790), L-histidine

monohydrochloride monohydrate (Sigma, H5659), L-isoleucine (Sigma, I7403), L-leucine (Sigma, L8912), L-lysine monohydrochloride (Sigma, L8662), L-methionine (Sigma, M5308), L-phenylalanine (Sigma, P5482), L-proline (Sigma, P5607), L-serine (Sigma, S4311), L-threonine (Sigma, T8441), L-tryptophan (L-TRP) (Sigma, T8941), L-tyrosine disodium dihydrate (Sigma, RES3156T-A7), L-valine (Sigma, V0513), Ro 61-8048 (Sigma, SML0233), AhR Antagonist II SR1 (Sigma, 182706), 2,3,7,8-tetrachlorodibenzo-p-dioxin (Supelco, 48599), nicotinic acid mononucleotide (Sigma, N7764) and N-formylkynurenine (NFK) (Toronto Research Chemicals, F700490). TRP-supplemented mTeSR1 medium was prepared through addition of L-TRP in 4-16-fold increments of the original concentration ($3.46 \times 10^{-2}$ mM) (Ludwig et al., 2006). Similarly, TRP-supplemented DMEM for HeLa and HEK293T cell lines was prepared by adding L-TRP incrementally based on the original concentration ($7.83 \times 10^{-2}$ mM). TRP-depleted custom DMEM medium was prepared through addition of 20 mM of D(+)-glucose (FUJIFILM Wako Pure Chemical, 049-31165), $2.26 \times 10^{-1}$ mM of bovine albumin fraction V Solution (Gibco, 15260037), Insulin ($3.44 \times 10^{-3}$ mM), Transferrin ($1.38 \times 10^{-4}$ mM), Selenium ($7.75 \times 10^{-5}$ mM) Solution (Gibco, 41400045), and 0.6 mM of L-Ascorbic Acid (Sigma, A5960) into glucose- and TRP-depleted DMEM medium kindly provided by Ajinomoto; TRP-replenished or 16-fold TRP-supplemented custom DMEM medium was prepared with $3.46 \times 10^{-2}$ mM or $5.54 \times 10^{-1}$ mM of L-TRP respectively. TRP-depleted StemFit medium was kindly provided by Ajinomoto; TRP-replenished AS103 medium was prepared with $3.46 \times 10^{-2}$ mM of L-TRP. Culture medium from the same lot was used for each experiment. Modified culture media were sterile filtered, and their volume was equalized to controls by adding DMSO (Sigma, D2650) and/or Milli-Q $H_2O$ where necessary. For acidic or basic reagents, pH of the solutions was adjusted using hydrochloric acid solution (Sigma, H9892) or sodium hydroxide solution (Sigma, S2770), and verified by Twin pH (Horiba). Solutions were stored in the dark at 4 °C, and if an original reagent required -20 °C storage, the solution prepared was divided into aliquots to minimize thaw-freeze cycles.

**Measurements of AA Consumption and Secretion of KYN and NFK**

Unused mTeSR1 medium overlying on a Matrigel-coated plate was sampled, and hiPSCs ($2.5 \times 10^5$) were then cultured in mTeSR1 with 10 μM of Y-27632 for 24, 48 or 72 h without medium change, and supernatant samples were stored at -80 °C until analysis. The fraction concentration of AAs as well as concentration of KYN in the consumed medium was measured with the LC-MS/MS system, as previously described (Shimbo et al., 2009; Tohyama et al., 2016). For measurement of KYN and NFK secretion, hiPSCs

($3.5 \times 10^5$) were cultured in mTeSR1 with 10 μM of Y-27632 for 2 days. Following removal of the supernatants, the cells were either left untreated or treated with TRP. Samples were collected every day and the media was changed every 48 h.

**Cell Proliferation Assays**

IncuCyte ZOOM (Essen Bioscience) was used for imaging assessment of cell proliferation by measuring cell confluence serially for specified periods. For hiPSCs or hESCs, $3 \times 10^4$ cells were plated in 6-well plates with the exception of assays using NFK, for which $1 \times 10^5$ cells were plated; while those including addition of inhibitors, Ro 61-8048 and SR-1, were plated at $2.5 \times 10^5$ cells; for AhR and IDO siRNAs, $2.5 \times 10^5$ cells were seeded. Alternatively, $2 \times 10^4$ HeLa cells and $1 \times 10^4$ HEK293T cells were seeded into 6-well plates. Cells were routinely incubated for 48 h in normal condition for stabilization, before transfer into medium consisting of compound to be tested, except for most of the inhibition assays mentioned above, where cells were incubated for 24 h. Cell confluence was calculated by imaging cells with whole-cell phase contrast imaging serially from the day of the initial medium change, using a lens apparatus and the following software recognition and analysis of cell confluence. For cell counts, Vi-CELL XR was used after specified periods of culture in a likewise manner.

**Alkaline Phosphatase Staining**

Plate wells were washed with D-PBS, and 4% paraformaldehyde fixative (Muto Pure Chemicals, 33111) was applied for 20 min before aspiration and washing with Milli-Q $H_2O$. A cocktail of FRV-Alkaline Solution, Sodium Nitrate Solution, Naphthol AS-BI Alkaline Solution of Leukocyte Alkaline Phosphatase Kit (Sigma, 86R), and Milli-Q $H_2O$ was added to the well according to the manufacturer’s instructions, and was shielded from light for 20–30 min prior to inspection.

**Immunocytochemistry**

Cells were washed once with D-PBS and fixed by adding 4% paraformaldehyde for 30-60 min. Cells were then washed twice more with D-PBS, and 0.1% Triton X was added for 1–15 min to permeabilize cells. Cells were then washed with PBS and blocked with ImmunoBlock (KAC, CTKN001) for 1 h or overnight. Following subsequent incubation with a primary antibody diluted in ImmunoBlock overnight at 4 °C, cells were washed twice with PBS, and co-incubated with a secondary antibody and 300 nM DAPI (Molecular Probes, D3571) for 2 h at room temperature in the dark. Finally, cells were washed twice with PBS and incubated in ImmunoBlock until staining was examined with

the inverted microscope Axio Observer.D1 (ZEISS) utilizing the accompanying software AxioVision (ZEISS). Primary antibodies used were: anti-OCT-3/4 (Santa Cruz, sc-5279; 1:200), anti-NANOG (ReproCELL, RCA0003P; 1:100), anti-SSEA4 (Chemicron, MAB4304; 1:200), anti-TRA-1-60 (Chemicron, MAB4360; 1:200), anti-IDO (Abcam, ab211017; 1:1000). Secondary antibodies used were: donkey anti-Mouse IgG (H+L), Alexa Fluor 488 (Molecular Probes, A-21202; 1:200), goat anti-Mouse IgM Heavy Chain Cross-Adsorbed, Alexa Fluor 488 (Molecular Probes, A-21042; 1:200), goat anti-Rabbit IgG (H+L) Cross-Adsorbed, Alexa Fluor 488 (Molecular Probes, A-11008; 1:200), and donkey anti-rabbit IgG (H+L) Highly Cross-Absorbed, Alexa Fluor 594 (Molecular Probes, A-21207).

**Flow Cytometry Analysis**

Dissociated single cells containing growth medium were centrifuged (300 g × 4 min) and dispensed into aliquot tubes with 100 μL of a solution containing 2% FBS in D-PBS, with 10 μL of the specific antibody added to each tube. The cells were left in the dark on ice for 30 min, and an additional 1 mL of solution was added before centrifugation (1,250 x g for 3 min). Supernatants were aspirated and after mixing of 500 μL of the added solution, cells were analyzed using a Gallios Flow Cytometer (Beckman Coulter). Antibodies used were: Anti-REA Control (S)-PE, human (Miltenyi Biotec, 130-104-612), Anti-SSEA-4-PE, human (Miltenyi Biotec, 130-100-635) and Anti-TRA-1-60-PE, human (Miltenyi Biotec, 130-100-350).

**Immunoblot Analysis for Protein Expression**

All procedures were performed in accordance to the manufacturer's instructions. Cell lysates were prepared by addition of a mixture of NuPAGE LDS Sample Buffer (Invitrogen, NP0007), NuPAGE Sample Reducing Agent (Invitrogen, NP0009), and Milli-Q $H_2O$ at a ratio of 5:2:13, followed by cell scraping and serial homogenization with an ultrasonic disruptor UR-21P (TOMY). Whole protein quantification was performed with a Qubit 3.0 (Thermo Fisher). After 10 min of heating at 70 °C, a gel electrophoresis was performed with a NuPAGE 4-12% Bis-Tris Gel (Invitrogen, NP0321) in Mini Gel Tank (Life Technologies) filled with 5% NuPAGE MES SDS Running Buffer (Invitrogen, NP0002), and with a PowerEase 300W Power Supply (Invitrogen). Upon completion, separated proteins were transferred onto a membrane using a PVDF mini iBlot 2 Transfer Stack (Invitrogen, IB24002) and iBlot 2 Gel Transfer Device (Invitrogen, IB21001). The membrane was then blocked with Blocking One (Nacalai tesque, 03953) for 30 min, washed by Milli-Q $H_2O$ for 5 min, and incubated

with a primary antibody diluted in T-BST (Takara, T9142) with rotation for 1 h. The membrane was then buffered with T-BST for 5 min (repeated 3x), inoculated with a HRP-conjugated secondary antibody (Sigma) for 30 min, buffered by TBS-T for 5 min (repeated 3x), and washed with Milli-Q $H_2O$ for 2 min (repeated 2x) in rotation, prior to visualization by Chemi-Lumi One (Nacalai tesque, 07880) or SuperSignal West Femto Maximum Sensitivity Substrate (Thermo Fisher, 34095). Images were obtained by a luminescent image analyzer LAS-3000 (FUJIFILM) or iBright FL1000 (Thermo Fisher Scientific), and band intensities were quantified by ImageJ (NIH). Primary antibodies used were: anti-GAPDH (Ambion, AM4300; 1:4000–1:25,000), anti-IDO (Abcam, ab211017; 1:1000) and anti-AHR (Abcam, ab190797; 1:1000).

**siRNA Knock Down of Gene Expression**

Lipofection was carried out in accordance with the manufacturer's instructions. At the time of medium change, a lipid-siRNA complex mixture of 9 µL of Lipofectamine RNA iMAX Reagent (Invitrogen, 13778) containing 150 µL of Opti-MEM (Gibco, 31985), and 25 pmol of siRNA with 150 µL Opti-MEM, was incubated for 5 min and added to a 6-well plate for analysis. siRNAs used were: Negative Control No. 1 Silencer (Ambion, A4611), IDO1 Silencer Select (Ambion, s7425) and AHR Silencer Select (Ambion, s1198).

**NAD/NADH Assay**

For individual quantification of NAD and NADH content, $2 \times 10^4$ cells/well were seeded onto a white 96-well plate (Corning, 3917), under normal growth conditions with 200 µL of mTeSR1 medium, and after 24 h wells were assigned into groups, where each group underwent a medium change with 200 µL of the specified medium. The cells were then incubated overnight before the NAD/NADH-Glo Assay (Promega, G9071) was performed, as per the manufacturer's instructions. Luminescence was scanned by EnSpire (Perkin Elmer).

**Metabolome Analysis**

hiPSCs 201B7 were cultured under normal maintenance conditions with mTeSR1 medium for 6 days and were either untreated or treated with TRP (for a final concentration of 553 µM) for 24 or 48 h. For the depletion study, after normal maintenance conditions cells were either treated with TRP-depleted StemFit or TRP-replenished StemFit medium for 6 h. For analysis of metabolites, methanol extraction was performed according to the Human Metabolome Technologies protocol. 201B7 cells were treated with 800 µL of

methanol for 30 s, and 550 μL of Milli-Q $H_2O$ containing internal standards (solution ID: H3304-1002, Human Metabolome Technologies, Inc., Tsuruoka, Japan) was added to the methanol extract. For analysis of medium from culture cells, 80 μL of supernatant was mixed with 20 μL of Milli-Q $H_2O$ containing the internal standards. The extract from the cells or medium was obtained and centrifuged at 2,300 × *g* at 4 °C for 5 min, and 700 μL of the upper aqueous layer was centrifugally filtered through a Millipore 5-kDa cutoff filter at 9,100 × *g* at 4 °C for 5 h to remove proteins. The filtrate was centrifugally concentrated and re-suspended in 50 μL of Milli-Q $H_2O$ for CE-MS analysis. Metabolome measurements were carried out through a facility at Human Metabolome Technologies Inc., Tsuruoka, Japan. CE-FTMS was carried out using an Agilent CE Capillary Electrophoresis System (Agilent Technologies, Waldbronn, Germany) equipped with a Q Exactive plus (Thermo Fisher Scientific Inc., Waltham, MA, USA), Agilent 1100 isocratic HPLC pump, Agilent G1603A CE-MS adapter kit, and Agilent G1607A CE-ESI-MS sprayer kit (Agilent Technologies, Waldbronn, Germany). The systems were controlled by Agilent G2201AA ChemStation software version B.03.01 for CE (Agilent Technologies, Waldbronn, Germany) and Xcalibur (Thermo Fisher Scientific Inc., Waltham, MA, USA). The metabolites were analyzed using a fused silica capillary (50 μm *i.d.* × 80 cm total length), with commercial electrophoresis buffer (Solution ID: H3301-1001 for cation analysis and H3302-1021 for anion analysis, Human Metabolome Technologies, Inc., Tsuruoka, Japan) as the electrolyte. The sample was injected at a pressure of 50 mbar for 10 s (approximately 10 nL) for cation analysis and 25 s (approximately 25 nL) for anion analysis. The spectrometer was scanned from *m/z* 60 to 900 for cation analysis and *m/z* 70 to 1,050 for anion analysis. Other conditions were as previously described (Sasaki et al., 2019; Soga and Heiger, 2000; Soga et al., 2003; 2002). Peaks were extracted using automatic integration software TraverseMS (Reifycs Inc., Tokyo, Japan) in order to obtain peak information including *m/z*, migration time for CE-FTMS measurement (MT), and peak area. The peaks were annotated with putative metabolites from the HMT metabolite database based on their MTs in CE and *m/z* values determined by FTMS. The tolerance range for the peak annotation was configured at ±0.5 min for MT and ±3 ppm for *m/z*. In addition, peak areas were normalized against those of the internal standards, and the resultant relative area values were further normalized by sample amount.

**Measurement of ECAR and OCR**

hPSCs ($1.2 \times 10^5$ cells) were seeded onto Matrigel-coated XF24 cell culture microplates (Seahorse Bioscience), cultured in mTeSR1 with 10 μM Y-27632, and incubated at 37 °C.

After ensuring sufficient confluency (~90%), medium was changed to glucose- and glutamine-depleted DMEM medium (Seahorse Bioscience) supplemented with 25 mM glucose, 1 mM pyruvate, 2 mM glutamine and 0.5% Insulin-Transferrin-Selenium Solution (Gibco, 41400045), and cells were grouped according to presence or absence of L-TRP (final concentration: $1.25 \times 10^{-1}$ mM for TRP-supplemented; $7.83 \times 10^{-2}$ mM for control). Conditioned and unconditioned cells were further incubated in a $CO_2$ free environment for 4 h, and OCR and ECAR were measured using a Mito Stress Kit (Seahorse Bioscience). Cells were analyzed using a XF24 Extracellular Flux Analyzer (Seahorse Bioscience) according to manufacturer's instructions.

**Statistical Analysis**

All statistical analysis was carried out using Prism (GraphPad). Ratio paired t-test, or unpaired t-test was employed for comparisons. Values are presented as means ± S.E.M; $^*p < 0.05$; $^{**}p < 0.01$; $^{***}p < 0.001$.

## SUPPLEMENTAL REFERENCES

Ludwig, T.E., Levenstein, M.E., Jones, J.M., Berggren, W.T., Mitchen, E.R., Frane, J.L., Crandall, L.J., Daigh, C.A., Conard, K.R., Piekarczyk, M.S., et al. (2006). Derivation of human embryonic stem cells in defined conditions. Nat. Biotechnol. *24*, 185–187.

Nakagawa, M., Taniguchi, Y., Senda, S., Takizawa, N., Ichisaka, T., Asano, K., Morizane, A., Doi, D., Takahashi, J., Nishizawa, M., et al. (2014). A novel efficient feeder-free culture system for the derivation of human induced pluripotent stem cells. Sci Rep *4*, 3594.

Sasaki, K., Sagawa, H., Suzuki, M., Yamamoto, H., Tomita, M., Soga, T., and Ohashi, Y. (2019). Metabolomics Platform with Capillary Electrophoresis Coupled with High-Resolution Mass Spectrometry for Plasma Analysis. Anal. Chem. *91*, 1295–1301.

Shimbo, K., Oonuki, T., Yahashi, A., Hirayama, K., and Miyano, H. (2009). Precolumn derivatization reagents for high-speed analysis of amines and amino acids in biological fluid using liquid chromatography/electrospray ionization tandem mass spectrometry. Rapid Commun. Mass Spectrom. *23*, 1483–1492.

Soga, T., and Heiger, D.N. (2000). Amino acid analysis by capillary electrophoresis electrospray ionization mass spectrometry. Anal. Chem. *72*, 1236–1241.

Soga, T., Ohashi, Y., Ueno, Y., Naraoka, H., Tomita, M., and Nishioka, T. (2003). Quantitative metabolome analysis using capillary electrophoresis mass spectrometry. J. Proteome Res. *2*, 488–494.

Soga, T., Ueno, Y., Naraoka, H., Ohashi, Y., Tomita, M., and Nishioka, T. (2002). Simultaneous determination of anionic intermediates for Bacillus subtilis metabolic pathways by capillary electrophoresis electrospray ionization mass spectrometry. Anal. Chem. *74*, 2233–2239.

Tohyama, S., Fujita, J., Fujita, C., Yamaguchi, M., Kanaami, S., Ohno, R., Sakamoto, K., Kodama, M., Kurokawa, J., Kanazawa, H., et al. (2017). Efficient Large-Scale 2D Culture System for Human Induced Pluripotent Stem Cells and Differentiated Cardiomyocytes. Stem Cell Reports *9*, 1406–1414.

Tohyama, S., Fujita, J., Hishiki, T., Matsuura, T., Hattori, F., Ohno, R., Kanazawa, H., Seki, T., Nakajima, K., Kishino, Y., et al. (2016). Glutamine Oxidation Is Indispensable for Survival of Human Pluripotent Stem Cells. Cell Metab. *23*, 663–674.
